# Insights Into the Clinical Learning Curve: Student Experiences in Prosthodontics Education

**DOI:** 10.7759/cureus.75589

**Published:** 2024-12-12

**Authors:** Naveed Inayat, Mahvish Wahad Khan, Nadia Munir, Mehvish Sajjad, Muhammad Moeed Haidar Naqvi, Abdullah Karamat, Dur E Shumyle

**Affiliations:** 1 Prosthodontics, Azra Naheed Dental College, The Superior University, Lahore, PAK; 2 Prosthodontics, Avicenna Medical and Dental College, Lahore, PAK; 3 Dental Materials, Avicenna Medical and Dental College, Lahore, PAK; 4 Dental Materials, University Dental College, The University of Lahore, Lahore, PAK; 5 Medical Education, Azra Naheed Dental College, The Superior University, Lahore, PAK

**Keywords:** active learning, clinical competence, knowledge application, learning strategies, medical education curriculum, patient care perception, practical skills, prosthodontic education, prosthodontics, self-efficacy

## Abstract

Background

The dental faculty must understand the challenges students face in prosthodontics to enhance education and meet patient care demands. This study explored final-year Bachelor of Dental Surgery (BDS) students' perceptions, study methods, and clinical application of knowledge, identifying gaps in translating theory to practice, skill acquisition, and curriculum alignment. Insights guide improvements in mentorship, hands-on training, and active learning to enhance clinical preparedness.

Materials and methods

This qualitative study was conducted on final-year BDS students using purposive sampling from six dental colleges in Lahore. Students answered semi-structured interview questions, while their responses were simultaneously documented by a trained transcriptionist. Data analysis was conducted via thematic analysis.

Result

The analysis revealed six themes: course difficulty and practicality, where theoretical concepts were understood but the practical application was difficult; study preparation, with varying time frames from early review to demanding last-minute study; distractions, including social media and peer interruptions; study resources, with textbooks and online tools as primary aids; active learning, generally encouraged but hampered by personal factors; and preparation for practice. The study data suggested that improving clinical practice and engagement techniques will enhance readiness.

Conclusion

This study concluded that the BDS course provided participants with good knowledge. However, the final-year BDS students consistently felt that their patient care abilities needed to improve in order to prepare them for future jobs.

## Introduction

Despite the advancements in dentistry and the ready availability of dental care, people still face tooth loss and require tooth replacement. The predoctoral prosthodontic courses that provide undergraduates with prosthodontic education are essential to meet society's dental therapeutic needs [1]. Most students are nervous about studying prosthodontics and fear not getting enough information from lectures regarding patient management and care, which leads to a lack of confidence when treating patients in the clinic [2].

Many students are concerned about which strategy to apply to study while preparing for an exam. Some of them prefer self-study, while others favor group learning. Carefully weighing the advantages and disadvantages of self-study and group study can maximize benefits and minimize drawbacks [3]. Evaluating the educational environment has become essential and is the need of the hour for both students and educators. A student’s capabilities, prior learning experiences, and knowledge are the aspects that determine a student's view of the learning strategy to be adopted by them [4]. Education now faces challenges in both theoretical and clinical settings. Educational planners must try to create an environment for students conducive to acquiring the necessary information and skills by making the best use of available resources [5].

Students need to emphasize performing clinical work on real patients, whereas faculty members should stress keeping their knowledge and skills in instructional methodologies on par with the current advancements [6]. To establish and improve educational programs, adequate educational planning is essential. A poorly designed and delivered course can have long-term consequences for students and other members of the community. As a result, the practical application of information and the provision of a suitable clinical context for teaching must be taken into account [7].

The goal was to assess the students’ perception of the subject of prosthodontics and to figure out their preferred instructional method. This will allow us to teach the students in the manner they want. This activity helped us understand students' psyches, which will help us in the future to teach them in ways appealing to them. With growing knowledge and understanding, some modifications will still be required for continuous improvement and achieving finesse. Provoking students to participate and engaging them in prosthodontic learning activities, ultimately enhancing fun learning to improve their interest, understanding, and clinical skills in dental clinics, should be the course of future teaching.

## Materials and methods

The study was conducted at Azra Naheed Dental College, The Superior University, Lahore, Punjab, Pakistan. This exploratory qualitative cross-sectional study was conducted after the approval of the Ethical Review Committee (Ref no. ANDC/RAC/2023/31) on final-year Bachelor of Dental Surgery (BDS) students, who were about to appear in their final professional exam. The study was conducted between July 15, 2023, and February 3, 2024, with the final interview completed on the latter date. Open-ended, semi-structured, face-to-face interviews were conducted using predetermined questions. The study included two public and four private dental institutes in Lahore, Punjab province. Students were asked to volunteer for the interview. Many students were willing, but we chose three students with different aptitudes representing each college's final-year class, resulting in a total of 18 participants. A student who scored more than 80% marks in the sendup was considered an excellent student, one with 70-80% was considered a good student, and one with 60-69% was categorized as average. Most students were excited to participate in this activity, although a few were a little anxious about this interview. Students were taken into confidence and briefly explained the purpose of this interview. Informed consent was taken, a confidentiality statement was mentioned, and the study objectives were explained. Students who achieved less than 60% were excluded from the research, as the emphasis was on exploring difficulties within the specified performance ranges. This classification facilitated an organized examination of different degrees of academic achievement, guaranteeing a varied representation of educational experiences and obstacles across a spectrum of skill levels. To ensure consistency and minimize interviewer bias, all interviewers received standardized training on the interview protocol, which included specific guidance on maintaining neutrality and consistency in questioning styles. Efforts were made to match interviewer demographics with the student population to foster a comfortable and relatable environment. Despite these precautions, subtle biases, such as differences in tone, non-verbal cues, or demographic factors like gender and ethnicity, might still exist, which could potentially influence the depth or nature of student responses. The interviews were conducted in a neutral and private setting within the departmental offices of the participating colleges, ensuring confidentiality and minimizing distractions. No faculty members or additional staff were present during the interviews to reduce external pressure on the participants. Students were given ample time to contemplate and respond to questions. Informed consent was obtained from all participants, and the purpose of the study was explained to alleviate anxiety and build trust. Each college recruited a prosthodontics department demonstrator, familiar with and trusted by the students, to conduct the interviews. The structured interview protocol ensured that key questions were consistently addressed across all interviews while still allowing flexibility for students to elaborate on their experiences. The data collected from these interviews was transcribed and analyzed thematically to identify key challenges and insights related to the clinical learning curve in prosthodontics.

## Results

The interviews were conducted to understand final-year BDS students’ perception and understanding of prosthodontics. A total of eighteen participants gave consent to be part of this study; the group included ten (55%) female and eight (45%) male students, representing diverse socioeconomic and educational backgrounds, among which six students (33.3%) were rated as excellent (achieving scores above 80%), eight (44.4%) were classified as good (scoring between 70-79%), and four (22.2%) were deemed average (with scores ranging from 60-69%) according to their sendup exam results. Of these participants, six students (33.3%) attended public institutions, while twelve (66.7%) were enrolled in private institutions in Punjab, Pakistan.

The feedback from students represented a range of responses and study methods in prosthodontics. Excellent students indicated that they started their preparation early, around three to six months before exams, and dedicated longer study periods compared to average students. Average students often initiated their study closer to the exam dates and took more frequent breaks between study sessions. The majority of students preferred studying alone, but group discussions became more common during the days of exam preparation, especially among good and average students.

In terms of resources, all groups majorly relied on using textbooks and lecture notes, but excellent students more frequently complemented their learning with additional online resources and artificial intelligence (AI) tools compared to their peers. Social media and peer distractions were the most mentioned obstacles to focused studying, with average students facing these challenges more significantly. Despite these variations, all groups agreed that it is the need of the hour to increase clinical exposure so that they can feel prepared for patient management and independent clinical practice.

This study found six themes that reflected students' experiences and obstacles in a prosthodontics course, directly linking these challenges to gaps in the current curriculum. Course difficulty and practicality discovered that, although theoretical principles were clear, practical application took time, necessitating much practice for clinical confidence. This gap underscores the need for curriculum reforms to integrate more hands-on training sessions and structured clinical exposure earlier in the learning process. Study preparation and strategies show a variety of techniques, with some students opting for steady, long-term study and others focusing on short-term-focused preparation. These findings suggest that the curriculum should incorporate flexible, individualized learning methods and promote consistent study habits through periodic assessments and guided study plans.

Distractions and concentration difficulties brought to light how social media, peer relationships, and psychological worries affect students' focus. Students' use of study materials revealed that they mostly depended on lecture notes and textbooks, supplementing their knowledge with additional internet resources such as videos and artificial intelligence technologies. Although active learning was encouraged, some students’ full participation was restricted by personal characteristics, including shyness and low self-esteem.

Lastly, students thought they required more clinical experience to feel confident inpatient treatment, which demonstrated a gap between theoretical understanding and practical competence in preparation for future practice. This finding highlights the need to align clinical teaching methods with real-world practice demands by increasing mentorship opportunities and simulated practice environments. Overall, students demonstrated flexibility in their use of resources and learning methods. However, they would still profit from more hands-on instruction, encouragement for concentrated study, and techniques to improve active learning engagement. These observations emphasize the necessity of modifying the curriculum to better prepare students for prosthodontic clinical practice (Table 1).

**Table 1:** Themes, sub-themes, and supporting quotes on student experiences in prosthodontic education AI: artificial intelligence

Theme	Short Definition	Description of Sub-Themes	Supporting Quotes
Course Difficulty and Practicality	Theoretical ideas in prosthodontics are easy to comprehend but difficult to implement, particularly in patient care.	Many students found the subject simple to learn theoretically but challenging to apply in clinical settings.	"Difficult to learn and very tricky. Easy to practice under proper guidance."
			"Subject is easy once you get the basics, but clinical work is essential to master it."
			"I like studying and love to do practical work, though it's difficult to understand without hands-on experience."
Study Preparation and Strategies	Exam preparation schedules differed; some students chose to study consistently and early, while others used rigorous, short-term studies closer to test dates.	Early Preparation: Students studied consistently over months before exams.	"I started preparing four months before."
		Short-Term Intensive Review: Others relied on focused study a few weeks or days before exams.	"I try to cover the stuff taught in class on the same day or week."
			"I initiated exam preparations approximately one week before exams."
Distractions and Focus Challenges	Social media, peer interactions, and personal anxieties were primary distractions during study time, reducing students' focus.	Social media, mobile devices, and external noise significantly impacted students' concentration during study sessions.	"Voices from around the house and neighbors' children playing were a distractor."
			"Social media notifications keep interrupting."
			"Mobile was a big distraction. I log out of all my social media accounts when I start preparing."
Use of Study Resources	Participants used a variety of resources, such as textbooks, lecture notes, and internet tools, to enhance their comprehension and promote classroom learning.	Primary Resources: Textbooks and lecture notes were the most used resources.	"I used recommended textbooks only."
		Supplementary Tools: Online videos, AI, and interactive platforms were also helpful.	"Textbooks, old papers, Google (Google LLC, Mountain View, California, United States), and ChatGPT (OpenAI, Inc., San Francisco, United States)."
			"Slides, reference books, and AI sometimes."
Active Learning Encouragement	Active learning was generally encouraged by faculty, but personal factors like shyness or lack of initiative limited some students' engagement.	Despite institutional encouragement, factors like shyness and reluctance limited students' active learning participation.	"Sometimes I’m encouraged, but I find it difficult to become active."
			"Yes, but I often find myself not taking it seriously."
			"I want to be an active learner, but I struggle with distractions and negligence."
Preparation for Future Practice	Although students obtained theoretical knowledge, several expressed a desire for more practical training and clinical experience in order to feel fully equipped for autonomous practice.	Students expressed greater confidence in academic knowledge than in actual skills and patient management. Many people mentioned a need for additional clinical practice.	"I’m not prepared yet; I still need confidence in problem-solving and clinical abilities."
			"The course provided basic knowledge, but independent patient management is still challenging."

## Discussion

This study was an excellent activity that helped us to understand the thinking of BDS final-year students and the problems faced by them during training. Moreover, the students were comfortable during the interview, and their views regarding different aspects of the BDS course were noted. Students are the finest people to judge the performance of any educational system. They often make reasonable comments to evaluate the teaching and evaluation techniques [8]. In the current study, information was gathered from students chosen to represent various aptitudes, and information was received from three distinct student categories. One typical practice is to distribute standardized questionnaires to students in order to determine the strengths and weaknesses of their educational system [9], but in this study, we chose to interview the students and gather qualitative feedback.

The current study found that the majority of students felt prosthodontics to be an easier subject theoretically but difficult to practice. Contrary to our result, another study reported it is difficult to learn theory in prosthodontics [10]. In yet another study it was reported that prosthodontics is considered a practical domain by both students and teachers, which necessitates active demonstrations and debates to master the practical aspects of the subject [11].

The top three study resources used by students were recommended textbooks, lecture notes, and the Internet. In another study, social media was reported to be used for dental learning by more than two-thirds of participants. The predominant advantages of using social media in learning were assistance in gaining more information on different subjects, making education more engaging, affording a better chance to access new resources, improving the ability for creativity and innovation, and improving research skills [12]. According to another study, the majority of respondents were reported to consider YouTube videos (Google LLC, Mountain View, California, United States) on clinical procedures to be a helpful learning tool. However, few of the respondents were uncertain about the evidence base of the videos [13].

According to a recent survey, students' preferred study approaches were "solo study," "group study," or a combo. While the most preferred location was a personal room. In this study, the majority voted for solo study and their own room as preferred choices for creating a study environment that is productive. Contrary to current study literature highlights, students’ preference is group discussion, as group discussions, along with integrated teaching approaches, serve to be the option for increasing student interest and learning outcomes [14]. Another study showed positive student perceptions of team-based learning pedagogy in terms of active engagement, knowledge acquisition, and improvement of interpersonal skills leading to more efficient learning outcomes [15]. Students preferred classroom learning for group discussion, as distance learning resulted in more difficult communication and gave less learning satisfaction. Less than half of the students preferred distance learning over classroom learning, although they agreed that distance learning was a more efficient learning method; it provided more time to study and review study materials [16].

The most common distraction during study preparation was social media, especially for private dental students. In another study, two-thirds of male students agreed that social media and mobile phones are distractions, while the majority of female students disagreed [17].

The current trend in dental education depends upon effective and active learning [18]. The majority of the students, especially from public dental institutes, expressed satisfaction with being encouraged to become active learners by their teaching faculty and institutes. According to a different survey, a large number of students were pleased with the acknowledgment, encouragement to ask questions, and the responses they received [14]. A study conducted in various dental institutions in Karachi found more than half of the participants received adequate attention from their lecturers to gain a concept and understanding of the subject [19].

When asked about the course contents, the majority responded that the course content and structure were according to their expectations. The workload was “moderate,” although the majority considered this subject practically challenging. The majority of the respondents reported that the faculty was cooperative. Subject in the BDS curriculum builds the foundation for upcoming practical learning [20]. The current study reported that the majority of the respondents, especially from private dental institutes, reported that the course was effective but practically difficult. Another study also reported the positive perception of students towards their education and learning, indicative of an overall favorable educational environment in the Department of Prosthodontics [21].

The dental students' knowledge, interpretation, and analytical skills are not retained by the didactic lecture without clinical sessions, which makes the subject dull and dry. Every lecture should be associated with and accompanied by a clinical application session [14].

The findings of this study align with existing literature on prosthodontics education, and it has revealed that prosthodontics theory is straightforward; it can be difficult to put into practice. This research further enhances our understanding by connecting these obstacles to particular deficiencies in the prosthodontics curriculum, including delayed engagement in clinical practice and the absence of formal mentorship programs. It establishes a foundation for curriculum enhancements that incorporate organized practical training and mentorship initiatives. These revelations seek to elevate educational results and equip students more effectively for clinical practice.

Strengths of the study

The study uncover gaps between theory and practice, offering vital insights for educators to enhance training methods. Involving students from six Lahore dental institutions adds depth and local relevance to prosthodontic education understanding. Highlighting student perspectives reveals often-overlooked insights in dental education research. This approach roots recommendations in real learner experiences, while thematic analysis uncovers shared challenges and diverse viewpoints. Addressing the scarcity of research on clinical learning in prosthodontics, this study opens avenues for future educational enhancements.

Limitations of the study

Potential interviewer bias affecting results despite standardization efforts. The focus on Lahore may limit the findings' broader applicability, while the purposive sampling might miss insights from underperforming or high-achieving students. These limitations warrant consideration in analyzing results, and future research could expand geographic reach and sampling methods.

## Conclusions

This study helped us understand the thinking of BDS final-year students and their training concerns. They have nearly the same attitudes, opinions, and thoughts with slight variance, such as preferring solo over group study, wishing to take notes and recollect them later during tests, being sidetracked by social media, and being encouraged to be active learners. However, they all agreed that while this course provided them with adequate knowledge, their patient management abilities need to improve in order to prepare them for future professional activities. 
